# Sex- and brain region-specific acceleration of β-amyloidogenesis following behavioral stress in a mouse model of Alzheimer's disease

**DOI:** 10.1186/1756-6606-3-34

**Published:** 2010-11-08

**Authors:** Latha Devi, Melissa J Alldred, Stephen D Ginsberg, Masuo Ohno

**Affiliations:** 1 Center for Dementia Research, Nathan Kline Institute, Orangeburg, New York 10962, USA; 2 Department of Psychiatry, New York University Langone Medical Center, New York, New York 10016, USA; 3 Department of Physiology and Neuroscience, New York University Langone Medical Center, New York, New York 10016, USA

## Abstract

**Background:**

It is hypothesized that complex interactions between multiple environmental factors and genetic factors are implicated in sporadic Alzheimer's disease (AD); however, the underlying mechanisms are poorly understood. Importantly, recent evidence reveals that expression and activity levels of the β-site APP cleaving enzyme 1 (BACE1), which initiates amyloid-β (Aβ) production, are elevated in AD brains. In this study, we investigated a molecular mechanism by which sex and stress interactions may accelerate β-amyloidogenesis and contribute to sporadic AD.

**Results:**

We applied 5-day restraint stress (6 h/day) to the male and female 5XFAD transgenic mouse model of AD at the pre-pathological stage of disease, which showed little amyloid deposition under non-stressed control conditions. Exposure to the relatively brief behavioral stress increased levels of neurotoxic Aβ42 peptides, the β-secretase-cleaved C-terminal fragment (C99) and plaque burden in the hippocampus of female 5XFAD mice but not in that of male 5XFAD mice. In contrast, significant changes in the parameters of β-amyloidosis were not observed in the cerebral cortex of stressed male or female 5XFAD mice. We found that this sex- and brain region-specific acceleration of β-amyloidosis was accounted for by elevations in BACE1 and APP levels in response to adverse stress. Furthermore, not only BACE1 mRNA but also phosphorylation of the translation initiation factor eIF2α (a proposed mediator of the post-transcriptional upregulation of BACE1) was elevated in the hippocampus of stressed female 5XFAD mice.

**Conclusions:**

Our results suggest that the higher prevalence of sporadic AD in women may be attributable to the vulnerability of female brains (especially, the hippocampus) to stressful events, which alter APP processing to favor the β-amyloidogenesis through the transcriptional and translational upregulation of BACE1 combined with elevations in its substrate APP.

## Background

Alzheimer's disease (AD) is a progressive neurodegenerative disorder and the most common cause of dementia in the elderly. One of the hallmark pathologies of AD is the senile plaque that is constituted of amyloid-β (Aβ) peptides. Although mutations in three different genes favoring the overproduction of Aβ are known to cause early-onset familial AD (FAD) [1,2], the etiology of sporadic AD that accounts for the majority of AD cases remains unclear. It is hypothesized that complex interactions between the genetic background and various environmental factors underlie sporadic AD [3,4] and a stressful lifestyle may represent one of the important risk factors for AD [5]. Elderly individuals prone to psychological distress are more likely to develop AD than those not prone to distress, and this trait is also associated with a more rapid progression of disease [6,7]. Consistent with the clinical observations, recent studies demonstrated that exposure to adverse behavioral stress accelerates the development of amyloid pathology and worsens memory decline in transgenic mouse models of AD [8-11], although the underlying molecular mechanisms have not been investigated in detail.

Meanwhile, it is also known that women have a higher risk of developing AD than do men. Although the longevity effect might be a factor in the preponderance of women with AD, the sex difference in AD prevalence remains even after age adjustment [12-14]. Interestingly, not only the sex but also the brain region represents a determinant of neuronal responses to stressful experience. For example, the hippocampus is a structure highly sensitive to stressor and is enriched with glucocorticoid receptors [15]. Furthermore, opposite effects of stress on hippocampal functions have been reported; an acute stressful event facilitates learning and increases dendritic spine density in male rats, whereas learning and spine density deteriorate after exposure to the same stressor in female rats [16-18]. Therefore, it is important to determine whether and how gender and adverse stress may interact to modify disease progression in different brain regions of AD transgenic mice.

The β-cleavage of amyloid precursor protein (APP) by BACE1 (β-site APP cleaving enzyme 1) initiates the generation of neurotoxic Aβ peptides. Notably, evidence is accumulating that increased levels of cerebral BACE1 and/or APP expression may be crucial contributing factors in developing sporadic AD [19-22]. In this study, we tested the hypothesis that behavioral stress may trigger the upregulation of BACE1 and/or APP, which may lead to differential acceleration of β-amyloidogenesis in the hippocampus and cerebral cortex of male and female subjects. Specifically, 5XFAD transgenic model mice at 3 months of age, which exhibit little or only faint amyloid pathology under normal conditions [23], were exposed to 5-day restraint stress, and we compared levels of BACE1, APP, its β-cleavage products and plaque burden between stressed and non-stressed subjects. We clearly demonstrate that the hippocampus of female 5XFAD mice shows the dramatic acceleration of β-amyloidogenesis with significantly elevated levels of both BACE1 and APP expression following the relatively brief stress treatment, providing a molecular basis for the higher prevalence and incidence of sporadic AD in women. Furthermore, our results also suggest that not only transcriptional but also translational mechanisms through phosphorylation of eukaryotic initiation factor-2α (eIF2α) may underlie BACE1 elevation associated with adverse stress during AD progression.

## Results

### Behavioral stress accelerates Aβ accumulation in 5XFAD mice in a sex- and brain region-dependent manner

**Figure 1:**
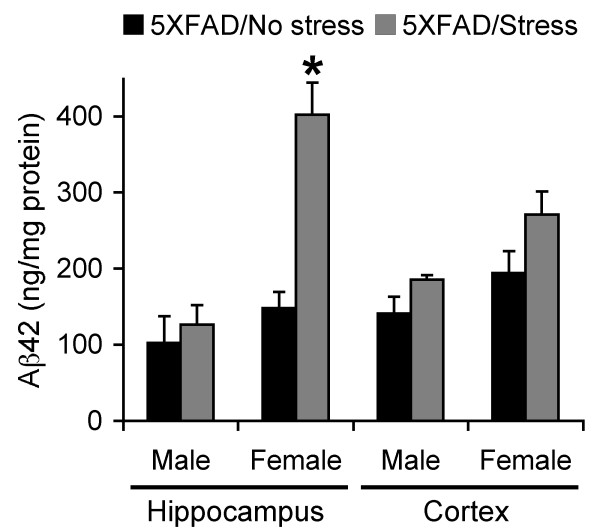
**Effects of behavioral stress on Aβ42 levels in the hippocampus and cerebral cortex of 5XFAD mice**. Male and female mice were exposed to restraint stress for 6 h per day during 5 consecutive days and were sacrificed for analysis 24 h after the last stress treatment. Levels of total Aβ42 were quantified by sandwich ELISA of guanidine extracts of hippocampal and cortical samples and expressed in nanograms per milligram of total protein (*n *= 5-8 mice per group). Note that Aβ42 levels are increased specifically in the hippocampus of stressed female 5XFAD mice as compared with non-stressed controls (*p < 0.05). All data are presented as mean ± SEM.

To examine the effects of adverse behavioral stress on the pathogenesis of AD, male and female 5XFAD mice at 3 months of age, which develop little or only slight amyloid deposition under normal conditions [23], were subjected to 5-day restraint stress (6 h/day). Twenty-four hours after the cessation of stress treatments, we first performed sandwich ELISA to compare Aβ levels between stressed and non-stressed 5XFAD mouse brains (Figure 1). The hippocampal and cortical samples were collected separately from male and female 5XFAD mice. The 5-day restraint stress significantly increased Aβ42 levels only in the hippocampus of female 5XFAD mice [*F*(1,14) = 29.00, *p *< 0.0001], while the other three groups of 5XFAD brain samples showed no significant difference in Aβ42 levels between stressed and non-stressed subjects (Figure 1).

**Figure 2:**
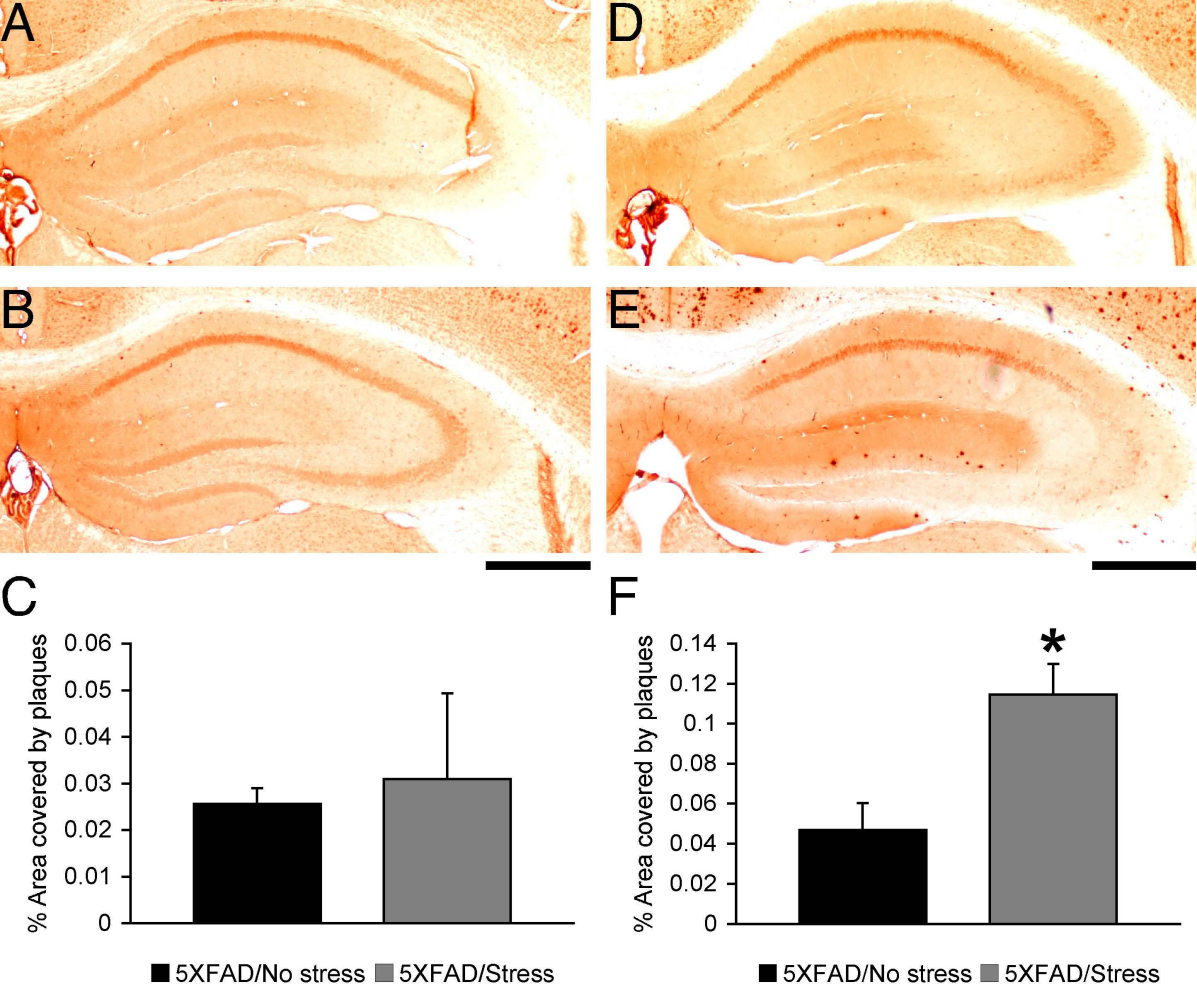
**Effects of behavioral stress on Aβ load in the hippocampus of 5XFAD mice**. Mice were exposed to restraint stress for 6 h per day during 5 consecutive days and were sacrificed for analysis 24 h after the last stress treatment. Brain sections from male (**A, B**) and female (**D, E**) 5XFAD mice were immunostained with the 6E10 anti-Aβ antibody. Shown are representative photomicrographs of the hippocampus of 5XFAD mice with (**B, E**) or without (**A, D**) stress treatments. Scale bar = 500 μm. Percentage area occupied by amyloid deposits in the hippocampus of male (**C**) and female (**F**) 5XFAD mice was measured for quantification (*n *= 6-7 mice per group). Note that hippocampal plaque burden is significantly increased in stressed female but not male 5XFAD mice as compared with non-stressed controls (* *p *< 0.05). All data are presented as mean ± SEM.

To test whether the ELISA measurements of Aβ42 reflected the acceleration of amyloid plaque formation, we conducted Aβ immunostaining using separate groups of male and female 5XFAD mice with or without stress treatments (Figure 2). Little or no amyloid deposition was found in the hippocampus of non-stressed male (Figure 2A) or female (Figure 2D) 5XFAD control mice. Importantly, plaque load, as measured by percentage area occupied by Aβ deposits, was significantly increased by exposure to the restraint stress in the hippocampus of female 5XFAD mice [*F*(1,12) = 10.96, *p *= 0.0062] (Figure 2E, F) but not in that of male 5XFAD mice (Figure 2B, C). In the cerebral cortex, non-stressed male and female 5XFAD mice exhibited faint amyloid deposition (mainly, in layer 5); however, stress treatments did not affect cortical Aβ burden in male or female 5XFAD mice (data not shown). Taken together, 5-day restraint stress elevated Aβ42 concentrations (~2.7-fold), leading to the higher plaque load (~2.4-fold) specifically in the hippocampus of female 5XFAD mice.

### Behavioral stress elevates BACE1 and APP levels in 5XFAD mice in a sex- and brain region-dependent manner

**Figure 3:**
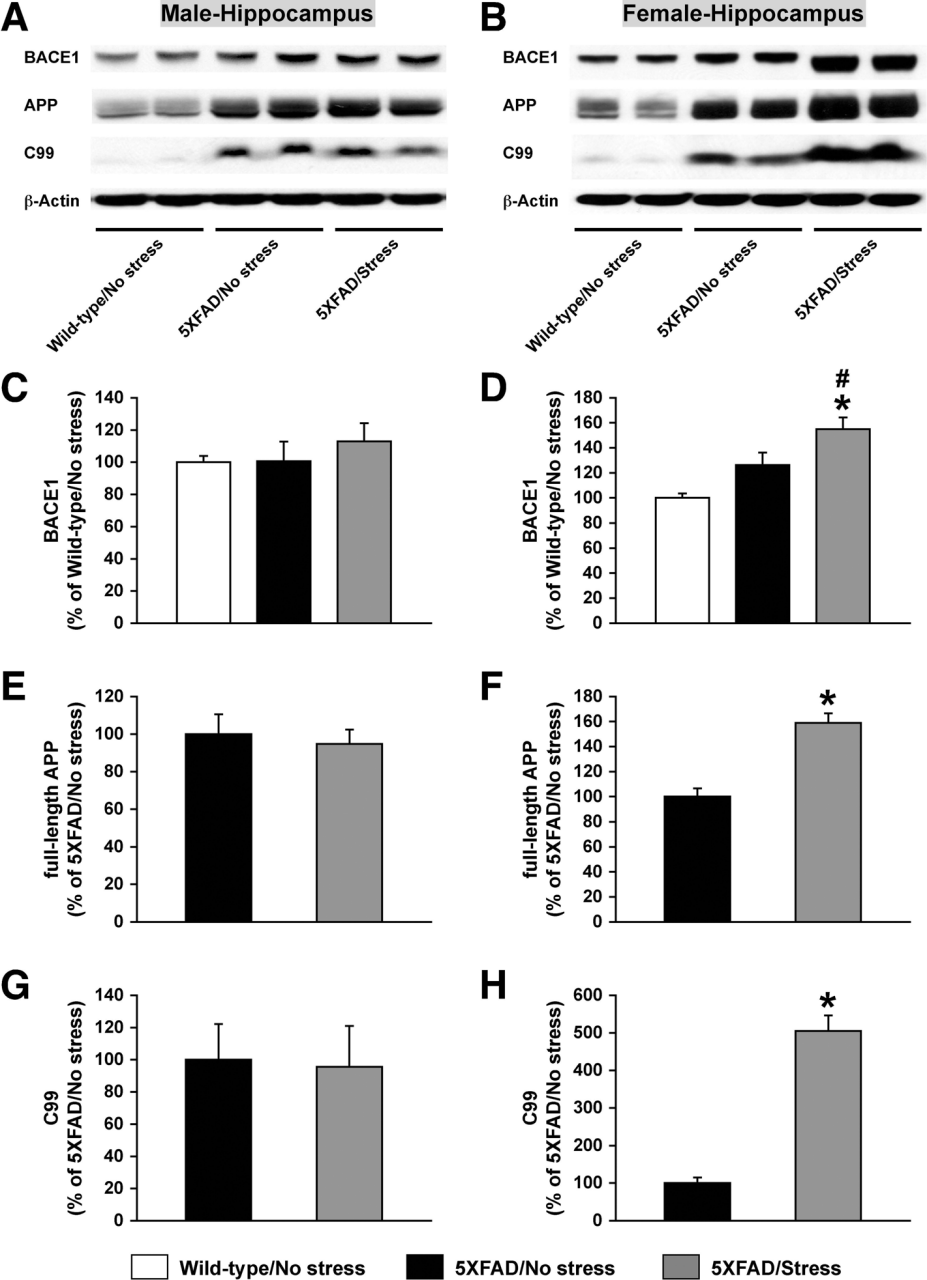
**Effects of behavioral stress on APP processing in the hippocampus of 5XFAD mice**. Mice were exposed to restraint stress for 6 h per day during 5 consecutive days and were sacrificed for analysis 24 h after the last stress treatment. Representative immunoblots of protein extracts from hippocampal homogenates of male (**A**) and female (**B**) 5XFAD mice with and without exposure to stress are shown along with those of non-stressed wild-type controls. Intensities of immunoreactive bands were quantified by phosphorimaging and were expressed as percentage of wild-type controls for BACE1 (**C, D**) or as percentage of non-stressed 5XFAD levels for full-length APP (**E, F**) and C99 (**G, H**). Data are presented as mean ± SEM for male (**C, E, G**) and female (**D, F, H**) subjects (*n *= 4-9 mice per group). ^#^*p *< 0.05 vs. non-stressed wild-type controls, *p < 0.05 vs. non-stressed 5XFAD mice.

To address the mechanisms underlying sex- and brain region-specific acceleration of Aβ accumulation following exposure to adverse stress in 5XFAD mice, we first compared stress-induced changes in hippocampal β-amyloidogenic APP processing between male and female 5XFAD mice (Figure 3). Immunoblot analysis of hippocampal homogenates from male (Figure 3A) and female (Figure 3B) mice demonstrated that 5-day exposure to restraint stress did not change BACE1 levels in male 5XFAD mice (Figure 3C), whereas it increased BACE1 expression in female 5XFAD mice (Figure 3D). A one-way ANOVA and *post-hoc *Fisher's PLSD test revealed that BACE1 levels in the hippocampus of stressed female 5XFAD mice were significantly higher than those of non-stressed wild-type and 5XFAD mice [*F*(2,18) = 10.40, *p *= 0.001]. Transgene-derived overexpression of human APP in 5XFAD mice was several folds relative to endogenous levels of APP detected in wild-type controls (Figure 3A, B). Similar to changes in BACE1 levels, the stress treatment significantly elevated levels of hippocampal full-length APP in female 5XFAD mice [*F*(1,14) = 32.47, *p *< 0.0001] (Figure 3F) but not in male 5XFAD mice (Figure 3E). In consequence, levels of the β-secretase-cleaved C-terminal fragment (C99) were not altered in the hippocampus of stressed male 5XFAD mice (Figure 3G), while C99 levels were dramatically elevated (~5-fold) in that of stressed female 5XFAD mice compared with non-stressed 5XFAD controls [*F*(1,14) = 67.21, *p *< 0.0001] (Figure 3H). We also tested how the same 5-day restraint stress would affect APP processing in the cerebral cortex of male and female 5XFAD mice. In contrast to changes observed in the hippocampus, the adverse behavioral stress did not significantly affect cortical BACE1 or full-length APP levels in male or female 5XFAD mice (data not shown). Collectively, 5-day exposure to restraint stress induced a marked increase of C99 levels, which was accompanied by significant elevations in both BACE1 and APP expression, specifically in the hippocampus of female 5XFAD mice consistent with the significantly increased Aβ42 concentrations and plaque burden (Figures 1 and 2)

### Transcriptional and translational mechanisms underlie sex- and brain region-dependent BACE1 elevation in 5XFAD mice following behavioral stress

**Figure 4:**
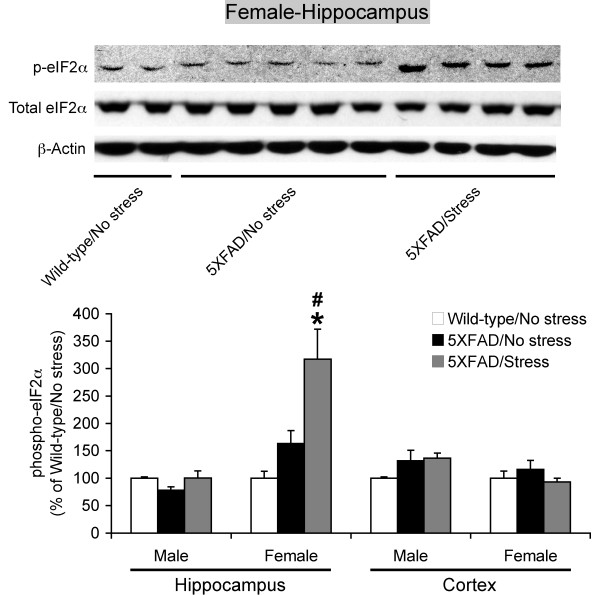
**Effects of behavioral stress on phospho-eIF2α levels in the hippocampus and cerebral cortex of 5XFAD mice**. Male and female mice were exposed to restraint stress for 6 h per day during 5 consecutive days and were sacrificed for analysis 24 h after the last stress treatment. Representative immunoblots of phosphorylated eIF2α (p-eIF2α) and total eIF2α for hippocampal homogenates of female 5XFAD mice with and without exposure to stress are shown along with those of non-stressed wild-type controls. Intensities of p-eIF2α-immunoreactive bands were quantified by phosphorimaging and were expressed as percentage of wild-type controls (*n *= 3-9 mice per group). Note that p-eIF2α levels, but not total eIF2α levels, are increased specifically in the hippocampus of stressed female 5XFAD mice as compared with non-stressed wild-type controls (^#^*p *< 0.05) and non-stressed 5XFAD mice (*p < 0.05). All data are presented as mean ± SEM.

**Figure 5:**
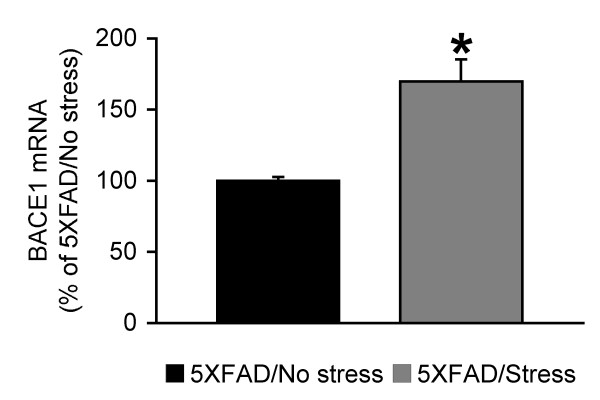
**Effects of behavioral stress on BACE1 mRNA levels in the hippocampus of female 5XFAD mice**. Mice were exposed to restraint stress for 6 h per day during 5 consecutive days and were sacrificed for real-time qPCR analysis 24 h after the last stress treatment. BACE1 mRNA levels were significantly elevated in the hippocampus of stressed female 5XFAD mice as compared with that of non-stressed 5XFAD controls (*p < 0.05) (*n *= 4 mice per group). All data are presented as mean ± SEM.

Recent evidence suggests that phosphorylation of the translation initiation factor eIF2α (phospho-eIF2α) plays an important role in mediating the post-transcriptional upregulation of BACE1 in sporadic AD and 5XFAD mouse brains at advanced stages of disease that develop massive amyloid pathology [24-26]. We first examined whether the phospho-eIF2α pathway was involved in the behavioral stress-induced BACE1 elevation in 5XFAD model mice (Figure 4). The baseline levels of phospho-eIF2α in non-stressed 5XFAD mice at the pre-pathological stage were not significantly different from those of wild-type control mice irrespective of sex or brain regions. In parallel with changes in BACE1 expression, 5-day restraint stress significantly increased phospho-eIF2α levels without affecting total eIF2α levels in the hippocampus of female 5XFAD mice [*F*(2,22) = 8.70, *p *= 0.0016]. In contrast, the other three groups of 5XFAD brain samples showed no significant changes in phospho-eIF2α levels between stressed and non-stressed animals. Moreover, qPCR analysis revealed that BACE1 mRNA levels were also significantly higher in the hippocampus of female 5XFAD mice exposed to behavioral stress as compared with that of non-stressed controls [*F*(1,6) = 20.00, *p *= 0.0042] (Figure 5). Taken collectively, the results clearly indicate that both transcriptional and translational mechanisms through activation of the eIF2α phosphorylation pathway underlie the female hippocampus-specific elevation of BACE1 expression in response to behavioral stressors in the 5XFAD model.

## Discussion

Very few AD cases can be attributable to genetic causes, while the etiology of sporadic AD that constitutes the majority of AD cases remains unclear [3,4]. Nevertheless, most transgenic models of AD are created based on a simple genetic association between the rare inherited form of FAD and excessive Aβ production and do not encompass acquired characteristics [27-30]. Many non-genetic factors including age, lifestyle (e.g., daily stress and diets), medical history and education have been reported to contribute to increasing the risk for AD [31,32]. Epidemiological investigations also show gender differences in the incidence and prevalence of AD with females being at higher risk [12-14], although a biological foundation for gender differences remains to be determined. In this study, we applied a relatively brief behavioral stress to male and female 5XFAD transgenic model mice at the pre-pathological stage of disease that shows little or only faint amyloid deposition, and tested the hypothesis that sex and stress interactions may represent a key mechanism underlying sporadic AD and rendering women more prone to develop AD.

Our results clearly demonstrated that 5-day exposure to restraint stress increased levels of Aβ42 peptides, a pathogenic Aβ species that is more hydrophobic and has the propensity to assemble into neurotoxic oligomers and aggregates [33-35], in the hippocampus of female 5XFAD mice but not in male 5XFAD mice. Meanwhile, the same stress treatment did not significantly affect Aβ42 levels in the cerebral cortex of male or female 5XFAD mice. Accordingly, amyloid plaque formation was also accelerated specifically in the female 5XFAD hippocampus following restraint stress. Interestingly, previous studies applied much longer stress treatments (e.g., several months of immobilization and/or isolation) to APP transgenic mice and showed that such prolonged stress exposures resulted in elevated Aβ concentrations and pathology in both the hippocampus and cortex without distinction based on sex [8-11]. Therefore, it should be noted that only 5-day exposure to restraint stress was sufficient to significantly elevate levels of Aβ42 and plaque load in 5XFAD mice in this study. Of particular importance, such a brief stress treatment revealed that the hippocampus of females is vulnerable and prone to develop amyloid deposits in response to adverse behavioral stressors. These findings support the idea that the higher prevalence of sporadic AD in women may be, at least in part, attributable to the vulnerability of female brain, especially the hippocampus, to stress mechanisms that favor β-amyloidogenic processing of APP. This is also consistent with the observation that hippocampal Aβ deposition is one of the earliest features of AD [36].

What mechanisms underlie the sex- and brain region-specific acceleration of Aβ accumulation following brief stress exposure? Previous studies demonstrate a sex difference in stress effects and estrogens are known to potentiate the glucocorticoid secretion under stress conditions [37,38]. This mechanism may represent the key component of the stress response that promotes β-amyloidosis more profoundly in female 5XFAD brains. Alternatively, recent studies have started to reveal the involvement of gender-specific molecular signaling processes or sex chromosome gene expression in addition to estrogen effects in causing sex differences in neuronal function [39,40]. Further study is needed to address the precise mechanisms including interactions between sex hormones and different stress mediators linking with changes in neuronal amyloidogenic processing of APP associated with AD.

Importantly, we found that BACE1 expression was elevated specifically in the hippocampus of stressed female 5XFAD mice in accordance with increased levels of Aβ42 and plaque burden. It has been reported that protein and/or activity levels of BACE1 become elevated in brains of sporadic AD patients [19-21,41] and 5XFAD mice [26,42-44] as disease progresses into the severer pathological stage with established amyloid plaques. Recent studies including ours demonstrate that phosphorylation of the translation initiation factor eIF2α plays a critical role in mediating the post-transcriptional upregulation of BACE1 associated with AD [25,26]. Increases in phospho-eIF2α levels occur in brains of sporadic AD and advanced pathological stages of APP transgenic mice including the 5XFAD model [24-26,45,46] and are shown to correlate with BACE1 elevation [25,26]. In the present study, baseline phospho-eIF2α levels of non-stressed 5XFAD mice at the pre-pathological stage were not significantly different from those of wild-type control mice, while they were increased by 5-day restraint stress exposure specifically in the hippocampus of female 5XFAD mice in line with BACE1 elevation. Therefore, these results suggest that the phospho-eIF2α-dependent translational upregulation of BACE1 in response to behavioral stressors may represent an important molecular mechanism by which environmental factors initiate β-amyloidogenesis before significant Aβ deposition occurs during the early phase of sporadic AD. This hypothesis is strongly supported by our recent observation that the increase in phospho-eIF2α induced by Sal 003, a specific inhibitor of its phosphatase, elevates BACE1 levels in younger 5XFAD mice, which have not yet showed BACE1 upregulation at basal levels concomitant with only marginal increases in eIF2α phosphorylation [26].

In addition to the translational mechanism, transcriptional control of BACE1 may also be implicated in AD pathogenesis [47,48]. In this study, we showed the increase of BACE1 mRNA level in the hippocampus of stressed female 5XFAD mice compared with that of non-stressed controls, suggesting a possibility that transcriptional mechanisms may also contribute to the BACE1 elevation associated with adverse behavioral stress. Our results are consistent with the findings that the promoter region of BACE1 gene contains glucocorticoid responsive elements [49] and that glucocorticoid administration facilitates Aβ production possibly via increases in transcription of the BACE1 gene through this binding site [50]. Some studies with postmortem human brains report elevations in BACE1 mRNA levels associated with sporadic AD [21,51], while others show no changes in mRNA despite the increased levels of BACE1 activity and protein [22,52-54]. Therefore, the mechanisms underlying BACE1 elevation in the sporadic AD brain remain controversial, which may be accounted for by differences in complex environmental factors mainly responsible for the disease progression. Our mouse model study suggests that both transcriptional and translational mechanisms may underlie BACE1 elevations associated with adverse stress during the development of AD.

Intriguingly, we also found that stress-responsive increases in APP expression levels occurred only in the hippocampus of female 5XFAD mice. Therefore, it seems likely that elevations in both BACE1 and its substrate APP work cooperatively to enable dramatic increases in the intermittent β-cleaved C-terminal fragment C99 and the acceleration of Aβ42 production and plaque formation in the hippocampus of female 5XFAD mice following 5-day exposure to behavioral stress. Given that Aβ and C99 peptides are amyloidogenic and can induce synaptic failure, neurodegeneration and memory loss [34,55-59], behavioral stress-dependent elevations in both β-cleavage products through BACE1 and APP upregulation have important implications for the pathogenesis of sporadic AD and the progression of neuronal dysfunction. Our findings are in agreement with recent reports showing that exposure to stress-level glucocorticoids (daily injections of dexamethasone for 7-21 days) elevates both BACE1 and APP levels leading to accelerated C99 production and Aβ accumulation [50,60,61]. However, these pharmacologically induced stress responses seem more robust than the behavioral stress regimen applied in this study as the changes are observed in brains of male subjects including 3xTg-AD transgenic model mice and middle-aged wild-type mice or rats. In any case, our present study combined with others indicates that elevated levels of glucocorticoids found in sporadic AD brains [62-64] may not only be a consequence of the pathology but also play a causal role in triggering β-amyloidogenesis through BACE1 and APP elevations during earlier stages of disease progression.

## Conclusions

Our mouse model study clearly demonstrates that the responsiveness of brains (especially, hippocampal neurons) to stress conditions, which shift APP processing toward β-amyloidogenesis by upregulating BACE1 and its substrate APP, represents a crucial contributing factor in the development of sporadic AD and may account for a mechanism underlying the increased prevalence of women to develop AD. Moreover, our data also suggest that transcriptional and translational mechanisms may underlie BACE1 elevation in response to adverse stressors, supporting the idea that therapeutic interventions aimed at suppressing stress-related signaling pathways (e.g., reduction of glucocorticoids or eIF2α phosphorylation) may be beneficial for slowing down AD progression.

## Methods

### Animals

We used 5XFAD transgenic mice that co-overexpress FAD mutant forms of human APP (the Swedish mutation: K670N, M671L; the Florida mutation: I716V; the London mutation: V717I) and presenilin-1 (PS1: M146L, L286V) transgenes under transcriptional control of the neuron-specific mouse Thy-1 promoter (Tg6799 line) [23,65]. 5XFAD lines (B6/SJL genetic background) were maintained by crossing hemizygous transgenic mice with B6/SJL F1 breeders (Taconic, Hudson, NY). 5XFAD transgenic mice used were hemizygotes with respect to the transgene and non-transgenic wild-type littermate mice served as controls. Genotyping was performed by PCR analysis of tail DNA. All experiments were done blind with respect to the genotype of the mice, and were conducted with the approval of the Nathan Kline Institute Animal Care and Use Committee.

### Behavioral stress

Stressed 5XFAD mice were individually placed in a well-ventilated plastic tube (Diameter: 3.8 cm) (541-RR, Plas-Labs, Lansing, MI) and restrained for 6 h per day during 5 consecutive days. After each stress session, the mice were returned to their cage where they were housed in isolation with free access to food and water. Twenty-four hours after the last exposure to restraint stress, brain samples were collected. The timing of stress treatments was arranged so that 5XFAD mice were precisely at 3 months of age when sacrificed. Non-stressed control mice were kept group-housed in their home cage and were sacrificed for analysis at 3 months of age.

### Aβ42 ELISA

Sandwich Aβ ELISA was performed as described previously [66,67]. Briefly, each hemibrain sample was extracted in 8X cold 5 M guanidine HCl plus 50 mM Tris HCl (pH 8.0) buffer, and centrifuged at 20,000 g for 1 h at 4°C to remove insoluble material. Final guanidine HCl concentrations were below 0.1 M. Protein concentrations were determined by a BCA protein assay kit (Pierce, Rockford, IL). To quantitate total levels of cerebral Aβ42, supernatant fractions were analyzed by a well-established human Aβ42 ELISA kits (KHB3441, Invitrogen, Carlsbad, CA) according to the protocol of the manufacturer. Optical densities at 450 nm of each well were read on a VersaMax tunable microplate reader (Molecular Devices, Sunnyvale, CA), and sample Aβ42 concentrations were determined by comparison with the standard curves. Aβ42 concentration values were normalized to total brain protein concentrations and expressed in nanograms per milligram of total protein.

### Aβ immunohistochemistry

Mice were transcardially perfused with 4% paraformaldehyde in phosphate buffered saline (PBS) under deep isoflurane anesthesia. The brain was removed and sectioned coronally at 40 μm on a vibratome (VT1200, Leica Microsystems, Wetzlar, Germany), and successive sections were stored in PBS containing 0.01% sodium azide at 4°C. Four sections per mouse were stained by the avidin-biotin peroxidase complex method for immunohistochemical analysis of amyloid deposition in the hippocampus and cerebral cortex [66,67]. Each section was separated by ~120 μm and taken at levels of -1.5/-1.9 mm to bregma according to the mouse brain atlas of Franklin and Paxinos [68]. The sections were incubated overnight at 4°C with monoclonal anti-Aβ1-16 antibody (1:200; 6E10, Signet, Dedham, MA). The ABC kit (PK-2200, Vector Laboratories, Burlingame, CA) was utilized with 3,3'-diaminobenzidine tetrahydrochloride as a chromogen to visualize the reaction product. The sections were then mounted on charged slides, dehydrated in a series of alcohol, cleared in xylene, and covered with a coverslip. Light microscopy was conducted on an Axioskop 2 microscope equipped with an AxioCaM HRc digital camera (Zeiss, Munich, Germany) for capturing images. Semi-quantitative analysis was performed using AxioVision imaging software with the AutoMeasure module (Zeiss). Identified objects after thresholding were individually inspected to confirm the object as a plaque or not in a blinded manner. Percentage area occupied by Aβ deposits in the hippocampus and cortex was assessed bilaterally, and the average of the individual measurements from each mouse was calculated to compare plaque load between the stressed and non-stressed 5XFAD mice.

### Immunoblot analysis

Hippocampal and cortical samples were taken from the mice under deep isoflurane anesthesia and were snap-frozen for Western blot analysis [66,67]. Each sample was homogenized in 5 volumes of modified RIPA buffer containing 150 mM NaCl, 50 mM Tris HCl (pH 8.0), 1 mM EDTA, 1% IGEPAL, 0.5% sodium deoxycholate, 0.1% SDS and protease/phosphatase inhibitor cocktails (Calbiochem, La Jolla, CA), and centrifuged at 10,000 g for 10 min to remove any insoluble material. Protein concentrations were determined by a BCA kit (Pierce), and 20-50 μg of protein was run on 4-12% NuPAGE gels (Invitrogen) and transferred to nitrocellulose membrane. After blocking, membranes were probed with anti-BACE1 (1:1,000, MAB5308, Millipore, Billerica, MA), an antibody that recognizes C-terminal epitope in APP (1:1,000, C1/6.1, kindly provided by Dr. Paul Mathews, Nathan Kline Institute) to detect full-length APP/C-terminal fragments, anti-phospho-eIF2α(Ser51) (1:1,000, #3398, Cell Signaling Technology, Danvers, MA), anti-eIF2α (1:1,000, #9722, Cell Signaling Technology) or anti-β-actin (1:15,000, AC-15, Sigma, St. Louis, MO). They were then incubated with horseradish peroxidase-conjugated secondary IgG. Immunoblot signals were visualized by an ECL chemiluminescence substrate reagent kit (Pierce) and were quantified by densitometric scanning and image analysis using Quantity One software (Bio-Rad Laboratories, Hercules, CA).

### Real-time qPCR

qPCR was performed in triplicate on frozen hippocampal samples as described previously [69,70]. TaqMan qPCR primers were utilized for mouse BACE1 mRNA (Mm00478671_m1, Applied Biosystems, Foster City, CA) and the housekeeping gene glyceraldehyde-3-phosphate dehydrogenase (GAPDH, Mm99999915_g1, Applied Biosystems). Samples were assayed on a real-time qPCR cycler (7900HT, Applied Biosystems) in 96-well optical plates covered with optical adhesive film. Standard curves and cycle threshold were generated using standards obtained from total mouse brain RNA. The delta delta cycle threshold (ddCT) method was employed to determine relative gene level differences between stressed and non-stressed 5XFAD mice with GAPDH qPCR products used as a control, and expression levels were presented as percentage of non-stress controls. Negative controls consisted of the reaction mixture without input RNA.

### Statistical analysis

The significance of differences between the groups was determined by a one-way ANOVA and *post-hoc *Fisher's PLSD tests were performed when appropriate. Data were presented as mean ± SEM and the level of significance was set for *p *value less than 0.05.

## Competing interests

The authors declare that they have no competing interests.

## Authors' contributions

LD performed a majority of the experiments, analyzed the data and wrote the manuscript. MJA and SDG performed the qPCR experiment and analyzed the data. MO designed the experiments and wrote the manuscript. All authors read and approved the final manuscript.
